# Relationship between Anatomical Variations of Sinonasal Area and Maxillary Sinus Pneumatization

**Published:** 2019-07

**Authors:** Najmeh Anbiaee, Raziyeh Khodabakhsh, Ali Bagherpour

**Affiliations:** 1 *Oral and Maxillofacial Diseases Research Center, Mashhad University of Medical Sciences, Mashhad, Iran.*; 2 *Private Practices, Toronto, Canada. *; 3 *Dentistry Research Center, Mashhad University of Medical Sciences, Mashhad, Iran. *

**Keywords:** Anatomical variations, Computed tomography scan, Maxillary sinus volume, Pneumatization

## Abstract

**Introduction:**

Maxillary sinuses are among the largest paranasal sinuses with various shapes and volumes. The dimensions and volumes of maxillary sinuses play an important role in the surgical treatment plan. The higher levels of pneumatization of alveolar bone lead to the increase of odontogenic sinusitis which cause problems in dental implantation. Therefore, this study aimed to evaluate the relationship between maxillary sinus volume and pneumatization and anatomical factors.

**Materials and Methods:**

In this cross-sectional study, computed tomography (CT) images of the healthy maxillary sinuses of 199 adult patients were reviewed. Amira software was used for the measurement of sinus volume. Sinus pneumatization of the alveolar bone in coronal CT scan images in the posterior teeth areas was measured. Moreover, anatomical variations of the sinonasal region, such as nasal septal deviation, and size of the ostium were measured and recorded. The Kolmogorov-Smirnov test, the t-test, and the Pearson’s and Spearman's correlation coefficients were applied for data analysis.

**Results:**

According to the obtained results, the mean value of the maxillary sinus volume and the alveolar bone pneumatization were 15.54 mm^3^ and 3.54 mm, respectively. The mean value of the maxillary sinus volume was statistically higher among males than females (P<0.001). The prevalence of nasal septal deviation, concha bullosa, and maxillary sinus septa were 14.6%, 14.6% and 6% respectively. There were no association between anatomical factors, including the nasal septal deviation, the size of the ostium, concha bullosa, and maxillary sinus septa and maxillary sinus volume and pneumatization. P-value less than 0.05 was statistically significant.

**Conclusion:**

No correlation was observed between the anatomical variations of the sinonasal region and maxillary sinus volume and pneumatization.

## Introduction

Paranasal sinuses are complex anatomical structures that vary from person to person (1). Maxillary sinuses are one of the largest paranasal sinuses; however, their shape and volume are also significantly different. Face dimensions and anatomical variations in the mid-face region are factors affecting the maxillary sinus dimensions (2,3).

Environmental factors, such as tooth extraction, and some inflammatory infections, including sinusitis, may also be related to the size of these sinuses (4,5).The dimensions of the maxillary sinuses and pneumatization of the alveolar bone have an important role in jaw surgery, dental implantation, and other dental treatments (6,7,8). The linear dimensions of maxillary sinuses have been investigated in several studies (4,9,10). However, volumetric research has been less considered (11). 

With regard to the effects of maxillary sinus volume and pneumatization of alveolar bone in surgery and dental treatments, three-dimensional volumes of the maxillary sinuses were examined in relation to anatomical factors. 

## Materials and Methods

**Fig 1:**
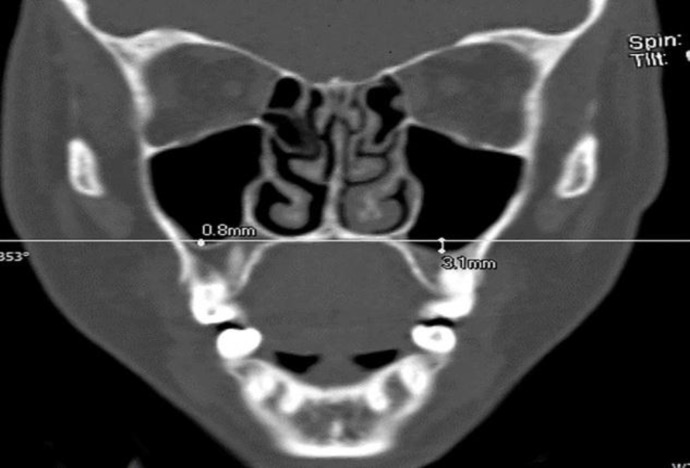
Measurement of alveolar bone pneumatization in posterior maxillary teeth area

Computed tomography (CT) images of the maxillary sinuses of 1,126 patients from the Radiology Department archive of Kamyab Hospital were reviewed. The CT scans of adult patients (over 18 years) were selected with no pathological features, such as sinusitis, polyps, fractures, cysts, and tumors. Totally, 199 cases who met the requirements of these conditions were included in the study. The Multislice Computed Tomography examinations were performed using a Sensation 16 scanner Siemens Medical Systems, Forchheim, Germany) with 2-5 mm slice thickness. In this study, linear dimensions, such as pneumatization of alveolar bone, middle nasal septal deviation, and maxillary sinus ostium size were measured in iQ-View (2.6.0) software. The alveolar bone pneumatization value was measured individually in millimeters regarding each posterior maxillary teeth. In the coronal view of CT scans, the tangential line of the floor of the nasal cavity was plotted. The distance between this line and the floor of maxillary sinus was measured as the amount of alveolar bone pneumatization (Fig.1).

If the maxillary sinus floor was above this line, pneumatization was marked with a negative sign. Otherwise, it was marked with a positive sign. With regard to the coronal view of CT scans, when the distance between the middle nasal septum and the midline was equal to or greater than 4 mm, it was considered as the nasal septal deviation (12). 

The maximum length of ostium was measured in millimeters, and the presence of concha bullosa and the number of maxillary sinus septa were recorded. All the measurements were taken by a dentist under the supervision of a maxillofacial radiologist.

**Fig 2:**
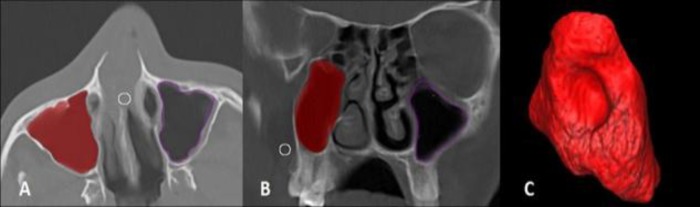
Surface area Calculation of Maxillary Sinus in axial (A) and Coronal (B) CT scans, 3D volumetric measurement of the maxillary sinus using the Amira software (C).

The Amira software (5.2.2), which Rae and Koppe used in their study, was applied to measure the volume of sinuses (13). Maxillary sinuses area was measured in both coronal and axial CT images (Fig.2). The volume of the sinuses was obtained separately in each view using the Amira 3D software. In addition, the mean volume of the sinuses was considered as maxillary sinus volume (Fig.2).

The data were analyzed in PASW Statisic 18. The Kolmogorov-Smirnov test, the t-test, the Pearson's correlation coefficient, and the Spearman's test were used to measure the data. P-value less than 0.05 were statistically significant.

## Results

In this cross-sectional study, 398 maxillary sinuses from 159 males and 40 females with the mean age of 30.36 (±14.71) and 32.65 (±13.22) years were evaluated, respectively (Table.1).

**Table 1:** Descriptive data about the age of patients

**Gender**	**N**	**Minimum**	**Maximum**	**Mean (SD)**	**95% C.I.**
Male	159	18	92	30.36 (14.71)	(28.04, 32.67)
Female	40	18	68	32.65 (13.22)	(28.42, 36.88)
Combined	199	18	92	30.82 (14.42)	(28.80, 32.84)

N: Number, I: Confidence interval, SD: Standard deviation

±±

The Spearman's correlation coefficient test showed a significant relationship between age and maxillary sinus volume (r<-0.15, P=0.03), indicating that the sinus volume decreases with age. The mean of sinus inferior pneumatization were 3.72 and 3.01 mm among males and females, respectively. However, the independent t-test showed that this difference was not statistically significant (P>0.05, Table.2).

**Table 2:** Descriptive findings of Maxillary Sinus Volume and Pneumatizaion

	**Gender**		**P-value**
	**Male **	**Female **	
	**Mean (95% CI)**		
LMSV*	15.44 (14.53, 16.36)	13.28 (11.97, 14.58)	0.008
RMSV*	15.77 (14.85, 16.69)	13.25 (12.09, 14.40)	0.001
LMSP⁑	3.37 (-5.84, 12.58)	2.64 (-4.32, 9.59)	0.36
RMSP⁑	4.00 (-5.72, 13.72)	3.41(-2.37, 9.19)	0.34

CI: Confidence interval, LMS: Left Maxillary Sinus Volume, RMS: Right Maxillary Sinus Volume, LMSP: Left Maxillary Sinus Pneumatization,RMSP: Right Maxillary Sinus Pneumatization.

* : cm^3^

⁑ : mm

Furthermore, the Pearson's correlation test showed a high correlation between the volume of the maxillary sinus and the alveolar bone pneumatization (P= 0.00 and r= 0.58 in right and r=0.72 in left).

Totally, 14.6% of the cases had concha bullosa on the right, left, or both sides. Concha bullosa was observed in 17.5% of females and 13.8% of males. The Chi-square test showed that there was no relationship between gender and concha bullosa incidence (P=0.55). Moreover, there was no meaningful relationship between the presence of concha bullosa and maxillary sinus volumes and pneumatization on both sides (P>0.05).

The Kolmogorov-Smirnov test showed that the size of the right and left ostium did not follow a normal distribution (P=0.001). Therefore, the Spearman's correlation test was used. The findings showed that there was no significant correlation between the mean volume and pneumatization of the right and left maxillary sinus and the size of the ostium on the same side (P>0.05).

 Out of 398 sinuses, only 23 of them (6%) had intersinus septum in this study. The findings suggest that the presence or absence of maxillary sinus septa has no significant association with maxillary sinus volume and pneumatization on the right and left sides (P=0.18, P=0.38, P=0.06, P=0.2, respectively).

In total, 29 (14.6%) cases had nasal septal deviation. The mean septal deviation was obtained 4.88 millimeter. The independent t-test showed that the middle nasal septal deviation had no effect on sinus volume and pneumatization on the right or left sides (P=0.26 and 0.77, P=0.19 and 0.9, respectively).

The mean sinus volume in dentate maxillary quadrant (n=38) and posterior edentulous maxillary quadrant (n=291) were 15.4 and 14.8 ml, respectively. However, this difference was not statistically significant (P=0.41). Other maxillary quadrants were partially edentulous (n=69). 

## Discussion

This study investigated the relationship between maxillary sinus volume and pneumatization in terms of sinonasal anatomical variations. The examined anatomical variations included nasal septal deviation, the size of the ostium, the presence of intersinus septa, and concha bullosa. The reason for the selection of these variations was that they could affect the airflow velocity in addition to the volume and pneumatization of the sinus (14-16).

Ikeda (1996), Kim HY (2008), and Hyun Cho (2010) noted that the volume of the maxillary sinus could be reduced by inflammatory or infectious diseases. Therefore, the patients with no symptoms of sinusitis or inflammatory disease were only selected for this study (17,18,5).

± and Ikeda (1996) and a similar 3D measurement technique performed by Park (2010) and Hyun Cho (2010). 

With regard to the linear approach, maxillary sinus volume was estimated by measuring the largest anterior-posterior and upper and lower dimensions of the sinus. However, in volume approaches, total sinus volume was obtained with a higher accuracy using multiple software programs (1,5,11,17).

 In a study conducted by Sahlstrand-Johnson (2011), there were no significant association between the sinus volume and the age. However, there was a significant correlation between the maxillary sinus volume and the age in the studies carried out by Seok Hyun and Ariji (r= -0.33). In this study, the volume of maxillary sinus decreased with the increase of the age; however, the correlation was not statistically significant (r=-0.15). 

Ariji et al. believed that these changes in sinus volume were due to the changes in the size of the body skeleton (1, 19). In this study, there was no statistically significant difference between the mean volume of sinus in the dentate and edentulous maxillary quadrant. However, Miguel Velasco-Torres stated that the dimensions of the maxillary sinus were reduced by aging and tooth loss (20). On the other hand, Schriber believed that being edentulous had no impact on sinus dimensions (21). Only 6% of the samples in this study had intersinus septa, which was a considerably lower frequency than reported in other studies. Park, Krennmair and Bornstein MM observed intersinus septa in 27.7% and 66.5% of their cases, respectively (9, 22-24). 

Selcuka et al. stated that intersinus septa were more likely among patients who had pathological findings in their maxillary sinus mucosa (25). In a study performed by Bornstein MM, 63.6% of the samples had thickening of the sinus membrane (24). Therefore, the lower number of intersinus septa in this study could be attributed to the fact that none of the patients had maxillary sinus pathology.

The prevalence of concha bullosa ranged from 14 to 53% based on the CT scan assessments (19,26-28). In the present study, concha bullosa was found in 29 (14.6%) patients. U. L. Demir showed no association between the existence of concha bullosa and the volume of the maxillary sinus. The findings were consistent with the obtained results of the present study (29). 

However, Duran Karats stated that there was a moderate positive correlation between concha bullosa volume and maxillary sinus volume (27). Nevertheless, the volume of the concha bullosa was not estimated in the present study. 

According to the results obtained from the present study, nasal septal deviation had no significant effect on maxillary sinus volume and pneumatization. Kapusuz Gencer Z. et al. showed that maxillary sinus volumes tended to be higher at the contralateral side of the severe septum deviations (15). They described the septal deviation as mild, moderate, and severe forms and it was found that its severe type was related to the sinus volume on the opposite side. 

Furthermore, the patients with sinusitis were not excluded from the study. Therefore, this can affect the outcomes as previously stated (15,18). Gokhan Gocmen affirmed that the nasal septal deviation and the presence of concha bullosa did not play a definite role in sinus inferior pneumatization (30).

Anatomical variations are more likely to increase inflammatory infections (e.g., sinusitis) rather than affecting the maxillary sinus volume and pneumatization (31-33). In this study, the number of the male case was higher than that of the female subjects. Therefore, it is recommended to include a balance regarding the number of both males and females in future studies. 

## Conclusion

In conclusion, the findings demonstrated that there were no associations between the maxillary sinus volume and pneumatization and anatomical variations, such as the size of the ostium, nasal septal deviation, sinus septa, and concha bullosa.
